# c-Jun-mediated microRNA-302d-3p induces RPE dedifferentiation by targeting p21^Waf1/Cip1^

**DOI:** 10.1038/s41419-018-0481-5

**Published:** 2018-04-18

**Authors:** Chao Jiang, Ping Xie, Ruxu Sun, Xiantao Sun, Guohua Liu, Sijia Ding, Meidong Zhu, Biao Yan, Qinghuai Liu, Xue Chen, Chen Zhao

**Affiliations:** 1 Department of Ophthalmology, The First Affiliated Hospital of Nanjing Medical University, Nanjing Medical University, 210029 Nanjing, China; 2 Department of Ophthalmology, Children’s Hospital of Zhengzhou, 450053 Zhengzhou, China; 3 Department of Ophthalmology, Qilu Children’s Hospital of Shandong University, 250000 Jinan, China; 4 Department of Science and Technology, The First Affiliated Hospital of Nanjing Medical University, Nanjing Medical University, 210029 Nanjing, China; 5 Save Sight Institute, Discipline of Clinical Ophthalmology and Eye Health, The University of Sydney, Camperdown, NSW 2000 Australia; 6 Department of Ophthalmology and Vision Science, Eye & ENT Hospital, Shanghai Medical College, Fudan University, 200023 Shanghai, China; 7 Key Laboratory of Myopia of State Health Ministry (Fudan University) and Shanghai Key Laboratory of Visual Impairment and Restoration, 200023 Shanghai, China

## Abstract

Dedifferentiation of retinal pigment epithelium (RPE) cells and choroidal neovascularization (CNV) contributes to the pathogenesis of age-related macular degeneration (AMD). MicroRNAs (miRNAs) have crucial roles in AMD onset and progression. We thus aim to investigate the effects of miRNAs on RPE dedifferentiation and endothelium cell (EC) behavior, and analyze its downstream pathways. We have previously identified miR-302d-3p as the most downregulated miRNA signature along with RPE differentiation. Herein, in vitro study supported that miR-302d-3p induces RPE dedifferentiation typified by reduction of RPE characteristic markers, interrupts its phagocytosis, and promotes its migration, proliferation, and cell-cycle progression. c-Jun was identified as a potential upstream transcript factor for *MIR302D*, which might modulate RPE function by regulating miR-302d-3p expression. P21^Waf1/Cip1^, a cyclin-dependent kinase inhibitor encoded by the *CDKN1A* gene, was identified as a downstream target of miR-302d-3p. Our data suggested that p21^Waf1/Cip1^ could promote RPE differentiation, and inhibit its proliferation, migration, and cell-cycle progression. We also demonstrated that miR-302d-3p suppresses RPE differentiation through directly targeting p21^Waf1/Cip1^. In addition, the miR-302d-3p/*CDKN1A* axis was also involved in regulating tube formation of ECs, indicating its potential involvement in CNV formation. Taken together, our study implies that miR-302d-3p, regulated by c-Jun, contributes to the pathogenesis of both atrophic and exudative AMD. MiR-302d-3p promotes RPE dedifferentiation, migration, proliferation and cell-cycle progression, inhibits RPE phagocytosis, and induces abnormal EC behavior by targeting p21^Waf1/Cip1^. Pharmacological miR-302d-3p inhibitors are prospective therapeutic options for prevention and treatment of AMD.

## Introduction

Retinal pigment epithelium (RPE), located in the outer retina between photoreceptor outer segments and choroidal vessels, is a monolayer of pigmented cells essential for maintaining regular retinal functions^1^. The post-mitotic RPE cells are required to cope with high metabolic rates and protein synthesis, digest toxic metabolite generated from photo transduction, and function under highly oxidizing conditions, all of which make RPE cells vulnerable to premature death. Abnormal RPE behaviors have been implicated in causing many retinal disorders, including age-related macular degeneration (AMD)^2,3^. AMD is a leading cause for irreversible vision loss in people aged over 55, and can be further categorized into the atrophic and exudative forms^4^. RPE dysfunction and depletion have preliminary causative roles in both forms. Other than abnormal RPE functions, exudative AMD is also typified by choroidal blood vessels growing through the Bruch’s membrane toward retina (choroidal neovascularization; CNV). Bleeding of these vessels may cause acute vision loss^5^. By far, no efficient treatment has been raised for atrophic AMD. Although therapies targeting neovascularization, like intravitreal injection of anti-vascular endothelial growth factor (VEGF) agents and photodynamic therapy (PDT)^6–8^, have been developed for AMD, treatment resistance, and CNV recurrence have been observed in a non-negligible fraction of patients^9–11^. We have previously identified that RPE dedifferentiation, characterized by reduction of RPE specific proteins, is an early consequence of AMD^12^. Thus, elucidation of early initiating events originating RPE abnormalities, especially RPE dedifferentiation, could allow the development of clinical preventions and interventions for AMD. However, the precise mechanism underlying RPE dedifferentiation is still poorly understood.

MicroRNAs (miRNAs) are small non-coding regulatory RNA molecules ranging from 19 to 25 nucleotides. miRNAs usually regulate gene expressions by directly binding to particular sites in the 3′-untranslated region (3′-UTR) of targeted mRNAs^13–15^. Other factors, including miRNA’s competition with other miRNAs, their interactions with transcriptional factors and long non-coding RNAs, and epigenetic modifications, like DNA methylation, would further confine a complete elucidation into their clear roles. By far, over 2000 human miRNAs have been identified, which regulate the expressions of almost 60% of protein-coding mRNAs including key factors involved in multiple signaling pathways, and stabilize gene networks against aberrant fluctuations^16–18^.

MiRNAs are involved in many biological processes including development and differentiation^19^. We have previously used a microarray to identify most differentially expressed miRNA signatures along with the differentiation from human-induced pluripotent stem cells (hiPSC) to RPE cells^20^. Our array data suggested that miR-302d-3p is consistently downregulated along with the differentiation, which was further proved by real-time PCR^20^. MiR-302d-3p is the mature miRNA encoded by the *MIR302D* (MIM: 614599) gene, which is located on 4q25 and belongs to the highly conserved miR-302 family. MiR-302 family has been revealed to target many biological pathways, including epigenetic regulation and cell-cycle progression^21–23^. However, the role of miR-302s in RPE dedifferentiation and CNV formation is poorly understood. In the present study, we aim to reveal the effects of miR-302d-3p on RPE dedifferentiation and endothelium cell (EC) behavior, and analyze its downstream pathway, thus finding out potential therapeutic targets to interrupt this process.

## Results

### MiR-302d-3p triggers RPE dedifferentiation

**Fig. 1:**
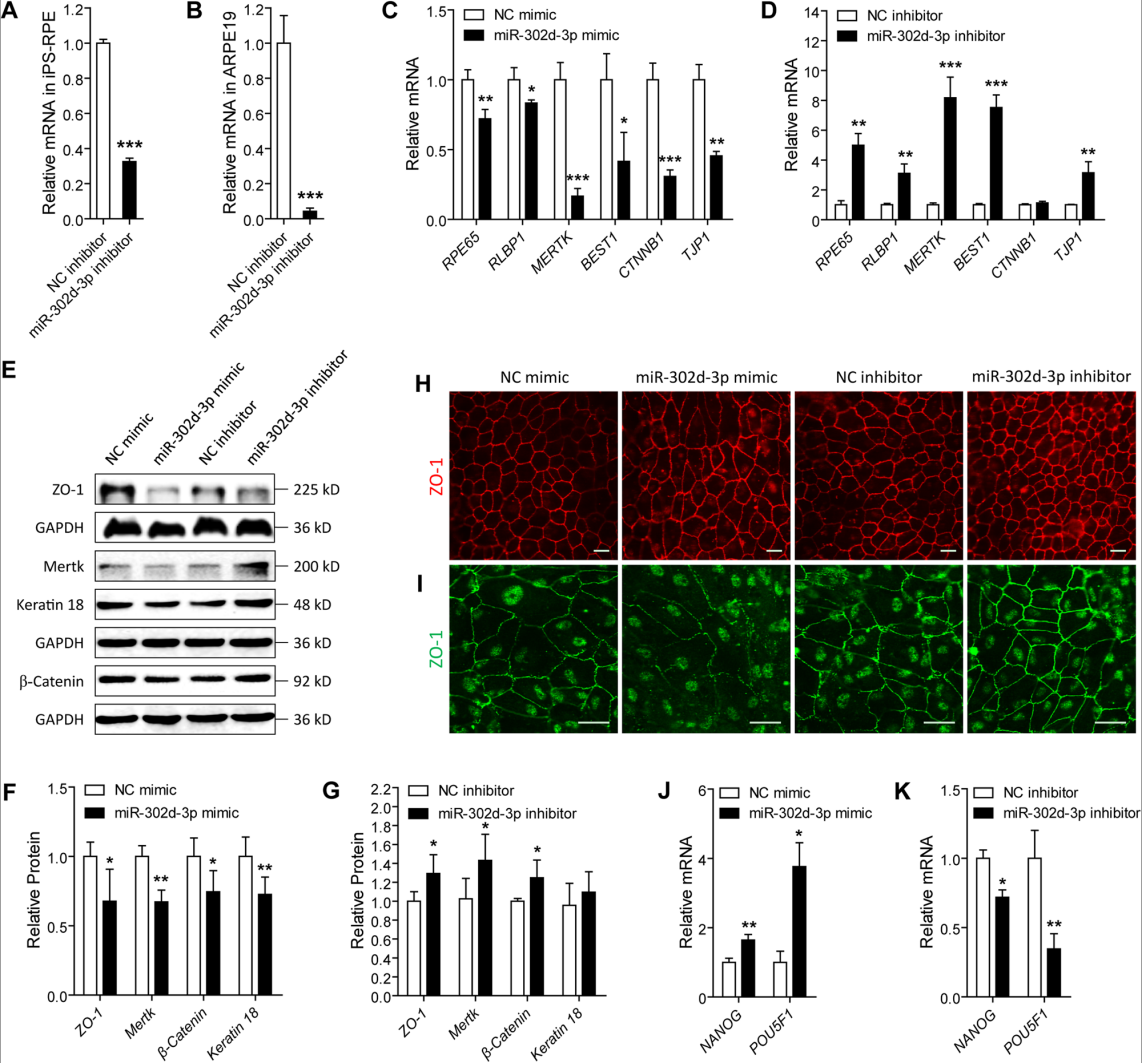
MiR-302d-3p inhibits RPE differentiation. **a**, **b** MiR-302d-3p expression was remarkably decreased in hiPSC-RPE cells at 30 dpd and ARPE-19 cells transfected with miR-302d-3p inhibitor compared to NC inhibitor. **c**, **d** Relative mRNA expressions of *RPE65*, *RLBP1*, *MERTK*, *BEST1*, *CTNNB1*, and *TJP1* were reduced in hiPSC-RPE cells at 30 dpd transfected with miR-302d-3p mimic compared to NC mimic (**c**), and were elevated in cells transfected with miR-302d-3p inhibitor compared to NC inhibitor (**d**). **e**–**g** Relative proteins of ZO-1, MERTK, β-Catenin, and Keratin 18 in hiPSC-RPE cells at 30 dpd transfected with NC mimic, miR-302d-3p mimic, NC inhibitor, and miR-302d-3p inhibitor. **h**, **i** Immunofluorescence revealed decreased expression of ZO-1 in hiPSC-RPE cells at 30 dpd (**h**) and ARPE-19 cells (**i**) transfected with miR-302d-3p mimic compared to cells transfected with NC mimic, while increased protein expression were detected in cells transfected with miR-302d-3p inhibitor compared to cells transfected with NC inhibitor. **j**, **k** mRNA expressions of pluripotency relevant genes *NANOG* and *POU5F1* were elevated in hiPSC-RPE cells transfected with miR-302d-3p mimic (**j**), and were reduced in cells transfected with miR-302d-3p inhibitor (**k**). *p < 0.05; **p < 0.01; ***p < 0.001. Scale bar = 20 μm

To investigate the role of miR-302d-3p on RPE differentiation, two cell lines, including hiPSC-RPE cells at 30 days post differentiation (dpd) and adult retinal pigmented epithelium (ARPE-19) cells, were transfected with miR-302d-3p mimic or inhibitor to modulate its expression. MiR-302d-3p mimic is chemically synthesized oligonucleotides identical to endogenous miR-302d-3p sequence, which could be loaded into RNA-induced silencing complex (RISC) and silence target genes like endogenous miR-302d-3p^24^. MiR-302d-3p inhibitors are antisense miR-302d-3p oligonucleotides, which could directly bind to the single strand mature miR-302d-3p to block its activity^25^. According to our results, endogenous miR-302d-3p expression was remarkably reduced in hiPSC-RPE and ARPE-19 cells transfected with miR-302d-3p inhibitor (Fig. 1a, b).

We initially compared expressions of RPE characteristic markers at the mRNA and protein levels among different transfected groups. mRNA of retinoid isomerohydrolase (*RPE65*; NM_000329), retinaldehyde-binding protein 1 (*RLBP1*; NM_000326), tyrosine-protein kinase Mer (*MERTK*; NM_006343), bestrophin-1 (*BEST1*; NM_001139443), catenin beta-1 (*CTNNB1*; NM_001904), and tight junction protein ZO-1 (*TJP1*; NM_003257) were measured by real-time PCR. Protein levels for ZO-1 (NP_003248), MERTK (NP_006334), keratin type I cytoskeletal 18 (encoded by *KRT18*, NM_000224; NP_000215), and β-Catenin (NP_001895) were determined via immunoblotting. Our results revealed that ectopic miR-302d-3p overexpression suppressed both the mRNA and protein expressions of RPE characteristic markers in hiPSC-RPE cells (Fig. 1c, e, f), while endogenous miR-302d-3p insufficiency promoted their expression (Fig. 1d, e, g). We next conducted immunofluorescence staining to show the expression pattern of ZO-1 in hiPSC-RPE and ARPE-19 cells. Consistently, ZO-1 expression was enhanced in both hiPSC-RPE and ARPE-19 cells with miR-302d-3p knocked down, but decreased in miR-302d-3p mimic transfected cells (Fig. 1h, i).

We next compared the mRNA expressions of two pluripotency relevant genes, including homeobox protein NANOG (*NANOG*; NM_024865) and POU domain class 5 transcription factor 1 (*POU5F1*; NM_002701). Our data revealed that both *NANOG* and *POU5F1* expression were elevated in hiPSC-RPE cells transfected with miR-302d-3p mimic (Fig. 1j), and were reduced in cells transfected with miR-302d-3p inhibitor (Fig. 1k). Taken together, our results indicated that miR-302d-3p induces RPE dedifferentiation.

### MiR-302d-3p inhibits RPE phagocytosis

**Fig. 2:**
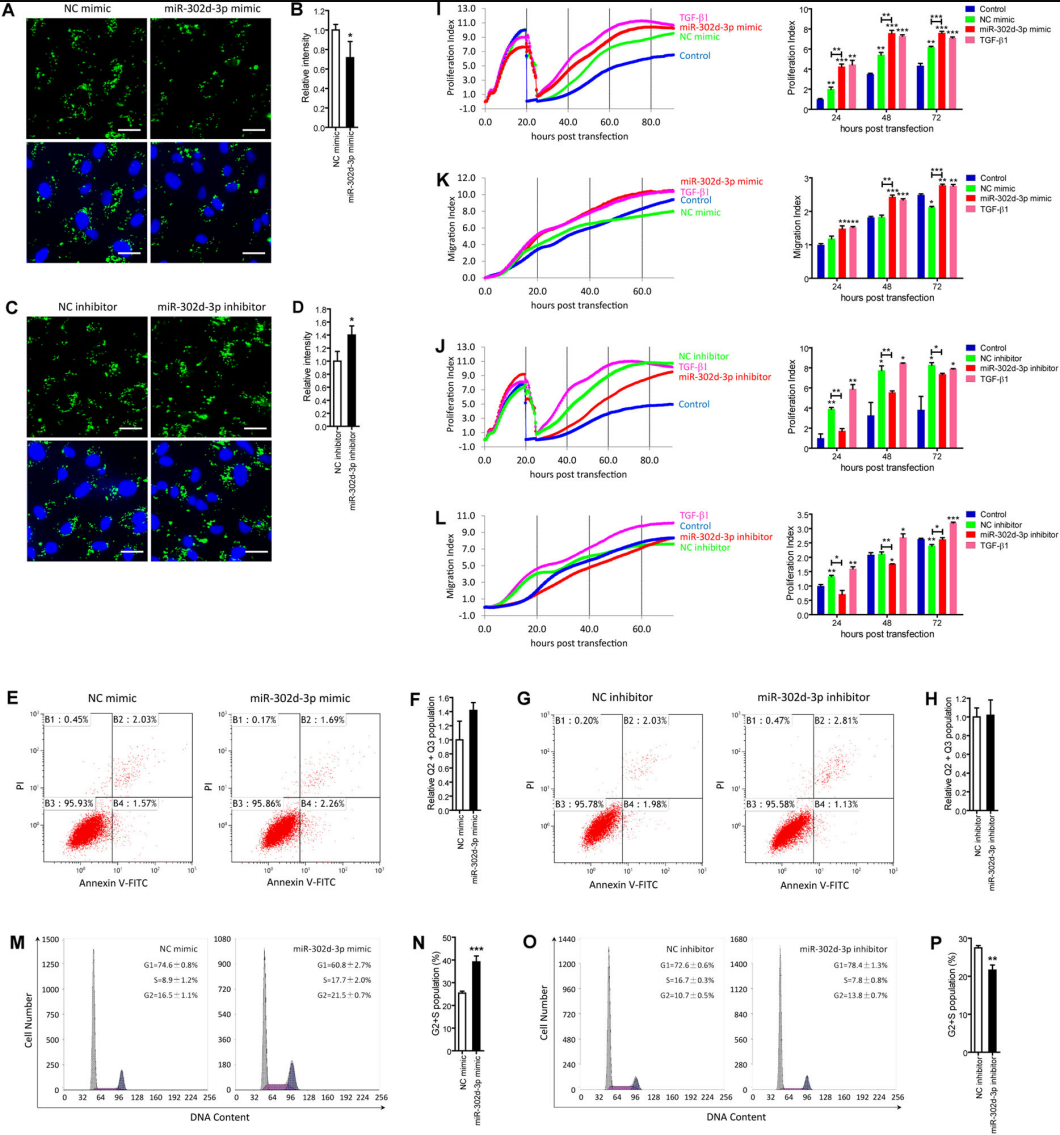
MiR-302d-3p disturbs RPE phagocytosis, promotes RPE proliferation, migration, and cell-cycle progression. **a**–**d** Phagocytosis ability was suppressed in ARPE-19 cells transfected with miR-302d-3p mimic compared to cells transfected with NC mimic (**a**, **b**), and was promoted in cells transfected with miR-302d-3p inhibitor compared to cells transfected with NC inhibitor (**c**, **d**). **e**–**h** No detectable change in apoptosis rate was revealed by Annexin-V-FITC/PI apoptosis assay in ARPE-19 cells transfected with miR-302d-3p mimic compared to cells transfected with NC mimic (**e**, **f**), nor in cells transfected with miR-302d-3p inhibitor compared to cells transfected with NC inhibitor (**g**,** h**). **i**–**l** Proliferative rates were elevated in ARPE-19 cells transfected with miR-302d-3p mimic compared to cells transfected with NC mimic (**i**), and were suppressed in cells transfected with miR-302d-3p inhibitor compared to cells transfected with NC inhibitor (**j**). Migration was promoted in ARPE-19 cells transfected with miR-302d-3p mimic compared to cells transfected with NC mimic (**k**), and was inhibited in cells transfected with miR-302d-3p inhibitor compared to cells transfected with NC inhibitor (**l**). **m**–**p** Cell-cycle progression was induced in ARPE-19 cells transfected with miR-302d-3p mimic compared to cells transfected with NC mimic (**m**,** n**), and was prohibited in cells transfected with miR-302d-3p inhibitor compared to cells transfected with NC inhibitor (**o**, **p**). Overall, 10,000 cells were analyzed for each sample. The values represented the percentage of cells in each phase of the cell cycle. *p < 0.05; **p < 0.01; ***p < 0.001. Scale bar = 20 μm

A crucial function of RPE is the phagocytosis of daily shed photoreceptor outer segments, which is essential to keep retinal homeostasis^26^. Impairment of RPE phagocytic ability has an essential role in AMD pathogenesis^27–29^. We next determined whether miR-302d-3p would disturb RPE phagocytosis. Consistent with above findings, phagocytic ability was suppressed in ARPE-19 cells transfected with miR-302d-3p mimic when compared to cells transfected with NC mimic (Fig. 2a, b), but was promoted in cells transfected with miR-302d-3p inhibitor compared to the NC inhibitor transfected group (Fig. 2c, d). To further determine whether the effects of miR-302d-3p on RPE phagocytosis is independent of RPE cell death, we next measured the role of miR-302d-3p on RPE apoptosis using the Annexin-V-FITC/propidium iodide (PI) apoptosis assay. According to our results, miR-302d-3p showed no detectable effect on the apoptosis of ARPE-19 cells (Fig. 2e–h), further supporting its role in interrupting RPE phagocytosis. Taken together, our data implied that miR-302d-3p inhibits RPE phagocytosis, thus interrupting regular RPE functions.

### MiR-302d-3p induces RPE migration, proliferation, and cell-cycle progression

Reportedly, dedifferentiation of post-mitotic tissues, including RPE, can be followed by cell proliferation and migration^19,30^. On the basis of our above findings, we hypothesized that miR-302d-3p might function in promoting RPE proliferation and migration. We continuously monitored cell proliferative and migratory rates up to 72 hours (h) post transfection to study the role of miR-302d-3p in ARPE-19 cells. Transforming growth factor beta-1 (TGF-β1) induces epithelial–mesenchymal transition in RPE cell, which could subsequently lead to its proliferation and migration^31^. Therefore, TGF-β1 treated ARPE-19 cells were taken as the positive control group, and untreated ARPE-19 cells were regarded as the negative control group. Both proliferation and migration were induced in cells overexpressing miR-302d-3p when compared to the negative control group and to cells transfected with NC mimic (Fig. 2i–k), and were inhibited in cells transfected with miR-302d-3p inhibitor when compared to the positive control group and to cells transfected with NC inhibitor (Fig. 2j–l). We next tried to determine the role of miR-302d-3p in cell cycle progression through DNA contents analysis. Our results demonstrated that miR-302d-3p overexpression increased the relative fraction of ARPE-19 cells in S and G2/M phases and decreased the fraction of ARPE-19 cells in G0/G1 phase (Fig. 2m, n), while silencing of miR-302d-3p showed opposite effects (Fig. 2o, p). Taken together, our findings suggested that miR-302d-3p could promote RPE migration, proliferation, and cell-cycle progression.

### c-Jun is a potential transcript factor of *MIR302D*

**Fig. 3:**
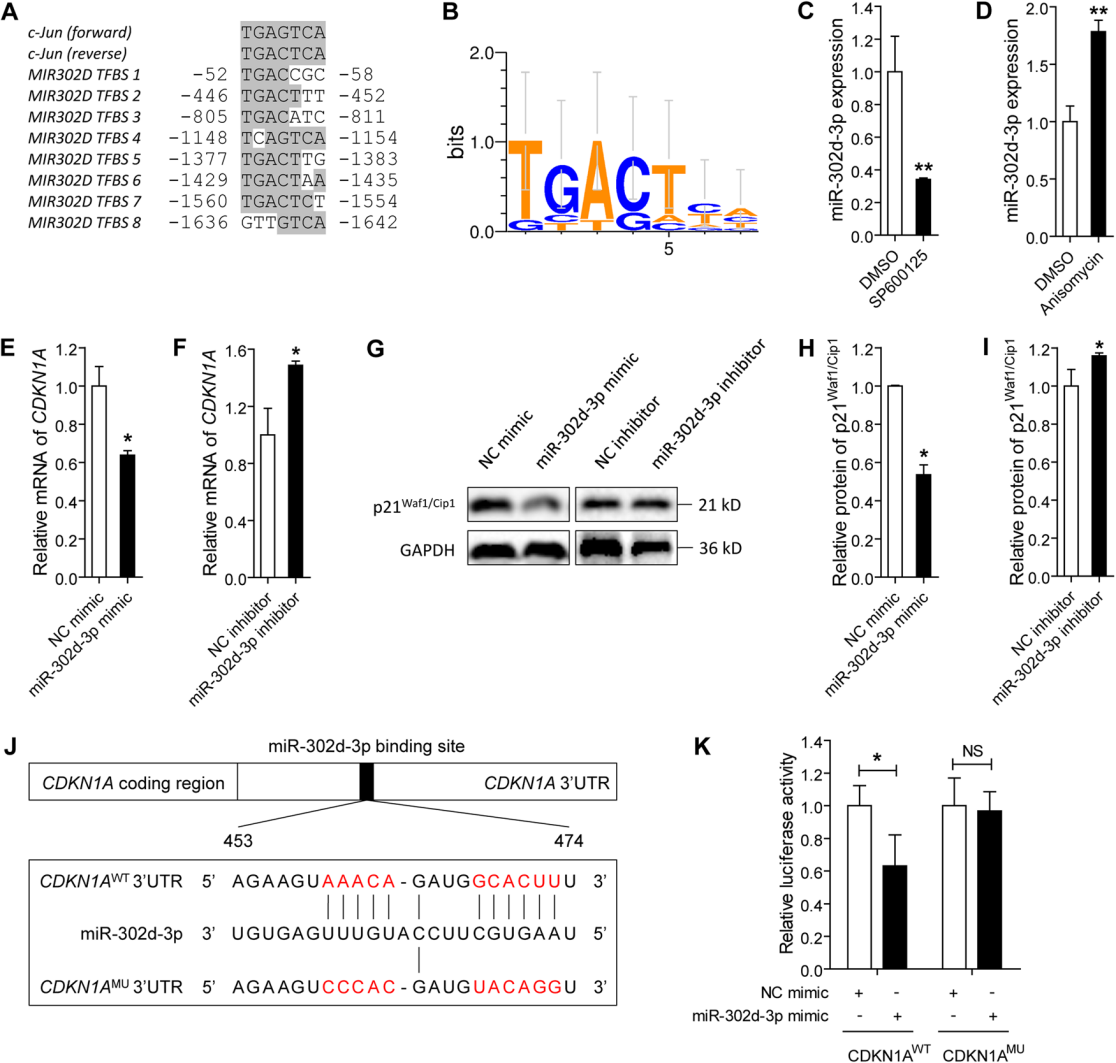
c-Jun-mediated miR-302d-3p targets the *CDKN1A* gene. **a** PROMO program predicts c-Jun as a putative transcript factor for *MIR302D* with 8 potential TFBS in its potential promoter region (1 to 2000 bp upstream of the transcription start site). **b** A sequence logo for all 8 TFBS in *MIR302D* promoter was created by WebLogo. **c** MiR-302d-3p expression was decreased in ARPE-19 cells treated with SP600125, a JNK inhibitor, compared to DMSO. **d** MiR-302d-3p expression was increased in ARPE-19 cells treated with Anisomycin, a JNK activator, compared to DMSO. **e**, **f** Relative mRNA expression of *CDKN1A* was suppressed in ARPE-19 cells transfected with miR-302d-3p mimic compared to NC mimic (**e**), and was elevated in cells transfected with miR-302d-3p inhibitor compared to NC inhibitor (**f**). **g**–**i** Relative protein of p21^Waf1/Cip1^ was downregulated in ARPE-19 cells transfected with miR-302d-3p mimic compared to NC mimic (**g**, **h**), and was upregulated in cells transfected with miR-302d-3p inhibitor compared to NC inhibitor (**g**, **i**). **j** Schematic diagram of the interaction between miR-302d-3p and *CDKN1A* 3′-UTR. **k** Relative luciferase activities were measured in ARPE-19 cells. Renilla luciferase vector was used as an internal control. *p < 0.05; **p < 0.01

PROMO online program (http://alggen.lsi.upc.es/cgi-bin/promo_v3/promo/promoinit.cgi?dirDB=TF_8.3) was applied to predict transcription factor binding sites (TFBS) in the potential promoter region (1 to 2000 base pairs [bp] upstream of the transcription start site) of the *MIR302D* gene (NR_029859)^32,33^. c-Jun was identified as a putative transcript factor of *MIR302D* with 8 potential TFBS in *MIR302D* promoter (Fig. 3a). Dissimilarities for the 8 TFBS were 10.15% (TFBS1), 5.78% (TFBS2), 7.54% (TFBS3), 4.44% (TFBS4), 6.67% (TFBS5), 3.81% (TFBS6), 3.24% (TFBS7), and 8.81% (TFBS8). A sequence logo for all 8 potential TFBS in *MIR302D* promoter was created by WebLogo (Fig. 3b)^34^. To better illustrate the regulatory role of c-Jun in *MIR302D* expression, we used SP600125 (Beyotime, Shanghai, China), an inhibitor of the c-Jun N-terminal kinase (JNK), and anisomycin, a JNK activator, to suppress or promote the JNK pathway and the activation of c-Jun. On the basis of our findings, expression of miR-302d-3p was remarkably reduced in ARPE-19 cells incubated with SP600125 compared to cells treated with dimethylsulfoxide (DMSO; Sigma, St. Louis, MO, USA) (Fig. 3c), while miR-302d-3p expression was elevated in ARPE-19 cells treated with anisomycin compared to DMSO treated cells (Fig. 3d). Thus, our study implies the regulatory role of c-Jun on *MIR302D* expression.

### *CDKN1A* is a direct target of miR-302d-3p in RPE cells

Cyclin-dependent kinase inhibitor 1 (*CDKN1A*; NM_000389) gene is previously found as a target of miR-302d in human adipose tissue-derived mesenchymal stem cells (hASDCs)^35^. We thus aimed to investigate whether *CDKN1A* is a target of miR-302d-3p in RPE cells. Initial assessments indicated that expression level of *CDKN1A* is inversely correlated with miR-302d-3p expression. In ARPE-19 cells overexpressing miR-302d-3p, both mRNA and protein levels of *CDKN1A* were decreased (Fig. 3e, g, h), while mRNA expression of *CDKN1A* were elevated in ARPE-19 cells transfected with miR-302d-3p inhibitor (Fig. 3f, g, i). Our data suggested that *CDKN1A* expression was inhibited by miR-302d-3p in both mRNA and protein levels. We next measured whether the effect of miR-302d-3p on *CDKN1A* expression is a direct consequence of miR-302d-3p binding *CDKN1A* 3′-UTR using luciferase reporter assay. We transfected ARPE-19 cells with miR-302d-3p mimic or NC mimic together with recombinant plasmids *CDKN1A*^WT^ and *CDKN1A*^MU^. *CDKN1A*^WT^ plasmid contains a 63 bp wild-type fragment of *CDKN1A* 3′-UTR covering binding sites with miR-302d-3p, while 11 nucleotides in the binding region were mutated in the *CDKN1A*^MU^ plasmid (Fig. 3j). According to our results, luciferase activity was significantly reduced in ARPE-19 cells co-transfected with *CDKN1A*^WT^ and miR-302d-3p mimic compared to cells co-transfected with *CDKN1A*^WT^ and NC mimic (Fig. 3k). Introduction of 11 nucleotides mutation located in the core binding region of *CDKN1A* abolished its ability to bind miR-302d-3p (Fig. 3k). Taken together, our findings suggested that miR-302d-3p directly targets *CDKN1A* 3′-UTR and suppresses *CDKN1A* expression in RPE.

### MiR-302d-3p modulates RPE differentiation by targeting p21^Waf1/Cip1^

**Fig. 4:**
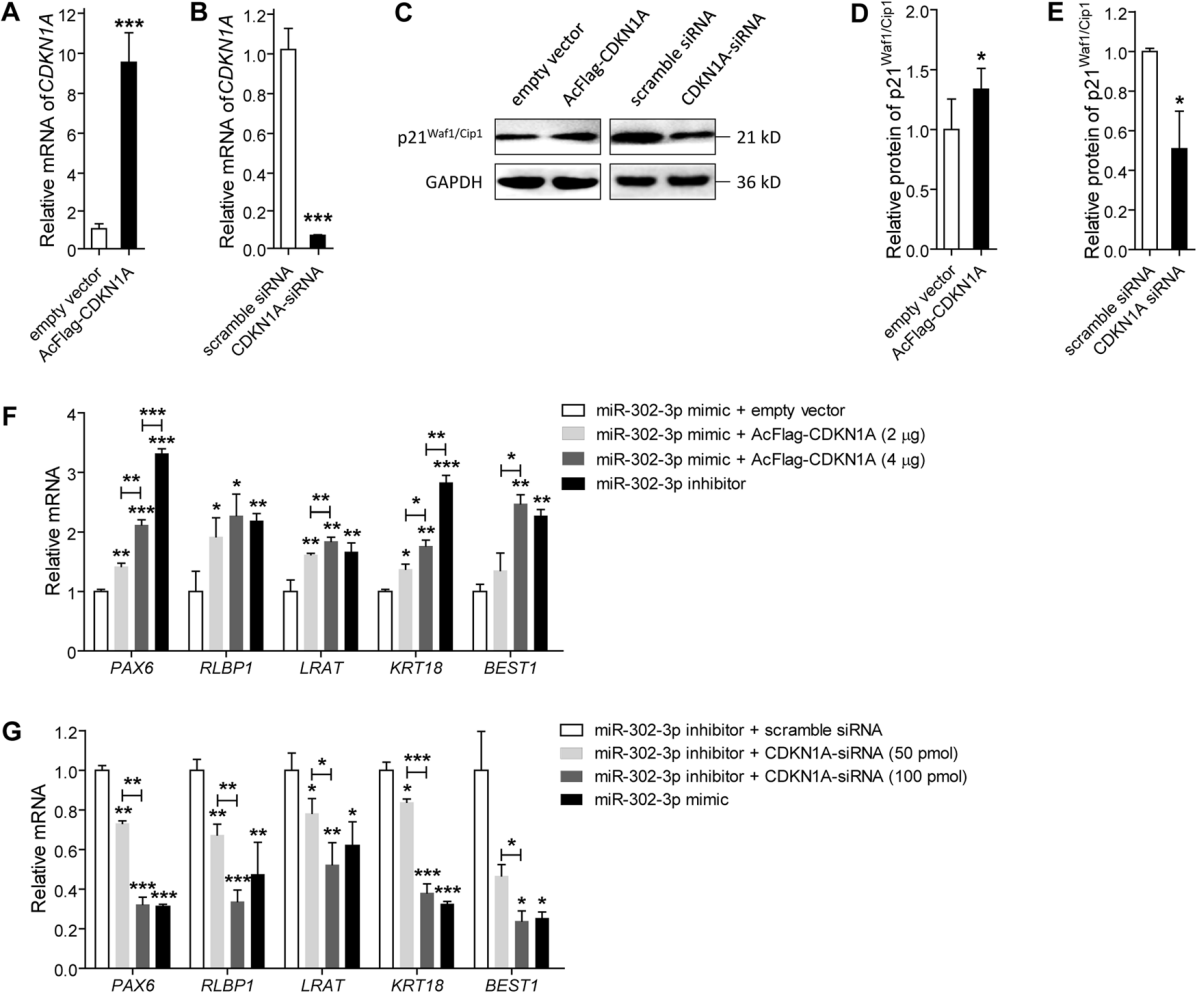
MiR-302d-3p regulates RPE function by targeting p21^Waf1/Cip1^. **a**, **b** mRNA expression of *CDKN1A* was increased in ARPE-19 cells transfected with AcFlag-CDKN1A compared to empty vector (**a**), and was decreased in cells transfected with CDKN1A-siRNA compared to scramble siRNA (**b**). **c**–**e** Protein expression of p21^Waf1/Cip1^ was upregulated in ARPE-19 cells transfected with AcFlag-CDKN1A compared to empty vector (**c**, **d**), and was downregulated in cells transfected with CDKN1A-siRNA compared to scramble siRNA (**c**, **e**). **f** mRNA expressions of *PAX6*, *RLBP1*, *LRAT*, *KRT18*, and *BEST1* in ARPE-19 cells transfected with miR-302d-3p mimic plus empty vector, miR-302d-3p mimic plus AcFlag-CDKN1A (2 μg), miR-302d-3p mimic plus AcFlag-CDKN1A (4 μg), and miR-302d-3p inhibitor. **g** mRNA expressions of *PAX6*, *RLBP1*, *LRAT*, *KRT18* and *BEST1* in ARPE-19 cells transfected with miR-302d-3p inhibitor plus scramble siRNA, miR-302d-3p inhibitor plus CDKN1A-siRNA (50 pmol), miR-302d-3p inhibitor plus CDKN1A-siRNA (100 pmol), and miR-302d-3p mimic. *p < 0.05; **p < 0.01; ***p < 0.001

P21^Waf1/Cip1^, protein encoded by the *CDKN1A* gene, is reported to prevent RPE cells from going through the G1/S phase checkpoint and inhibit their proliferation^36^. Thus, we hypothesized that miR-302d-3p modulates RPE differentiation by targeting p21^Waf1/Cip1^. We next detected whether p21^Waf1/Cip1^ could mediate the effects of miR-302d-3p on RPE differentiation. Effectiveness of AcFlag-CDKN1A and CDKN1A-small interfering RNA (siRNA) in modulating *CDKN1A* mRNA and p21^Waf1/Cip1^ protein expressions was initially confirmed in ARPE-19 cells (Fig. 4a–e). Noteworthy, three pairs of siRNA oligoes targeting different regions of *CDKN1A* were designed and synthesized (data not shown). Only siRNA with best efficiency and stability was selected for further investigations. AcFlag-CDKN1A presented a transfection efficiency of over 60% in both ARPE-19 cells and human umbilical vein endothelial cells (HUVECs). mRNA expressions of several RPE characteristic markers, including paired box protein pax-6 (*PAX6*; NM_001127612), *RLBP1*, lecithin retinol acyltransferase (*LRAT*; NM_004744), *KRT18*, and *BEST1*, were monitored in ARPE-19 cells from different transfected groups to assess RPE status. We found that overexpression of p21^Waf1/Cip1^ could mediate the negative effect of miR-302d-3p on RPE differentiation in a dose-dependent manner (Fig. 4f). In addition, with sufficient p21^Waf1/Cip1^ dosage, the negative effect could be fully rescued (Fig. 4f). Consistently, *CDKN1A* silencing was also found to suppress RPE differentiation in a dose-dependent manner (Fig. 4g). Silencing of *CDKN1A* could also fully disturb RPE differentiation (Fig. 4g). Collectively, our findings suggest that miR-302d-3p inhibits RPE differentiation via directly targeting p21^Waf1/Cip1^.

### P21^Waf1/Cip1^ promotes RPE differentiation, and inhibits RPE proliferation, migration, and cell-cycle progression

**Fig. 5:**
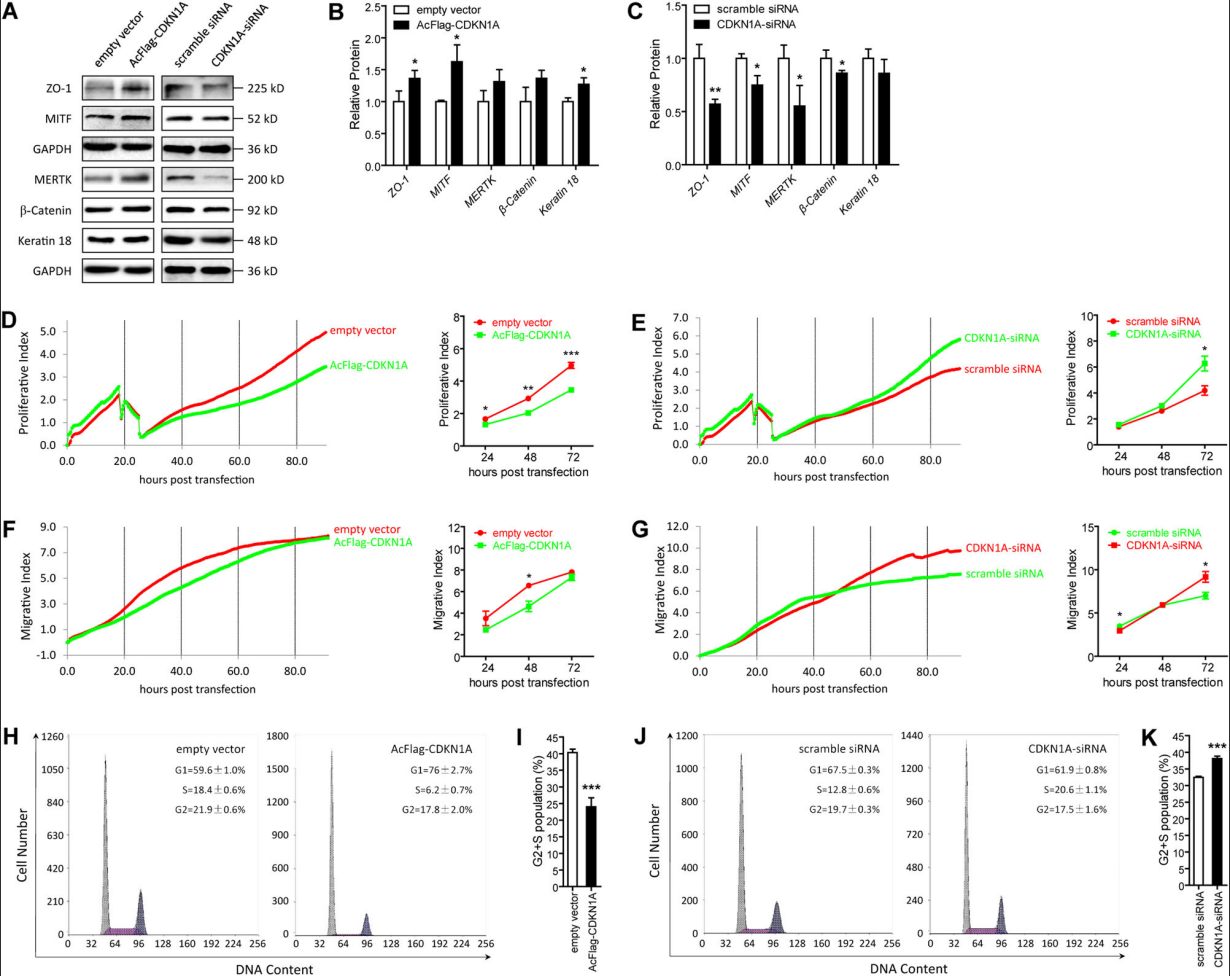
P21^Waf1/Cip1^ promotes RPE differentiation, and inhibits cell proliferation, migration, and cell-cycle progression. **a**–**c** Immunoblotting suggested that protein expressions of ZO-1, MITF, MERTK, β-Catenin, and Keratin 18 were increased in ARPE-19 cells transfected with AcFlag-CDKN1A compared to cells transfected with empty vector (**a**, **b**), and were decreased in cells transfected with CDKN1A-siRNA compared to cells transfected with scramble siRNA (**a**, **c**). **d**–**g** Proliferative rates were downregulated in ARPE-19 cells transfected with AcFlag-CDKN1A compared to empty vector (**d**), and were upregulated in ARPE-19 cells transfected with CDKN1A-siRNA compared to scramble siRNA (**e**). Migration was inhibited in ARPE-19 cells transfected with AcFlag-CDKN1A compared to empty vector (**f**), and was promoted in cells transfected with CDKN1A-siRNA compared to scramble siRNA (**g**). **h**–**k** Cell-cycle progression was suppressed in ARPE-19 cells transfected with AcFlag-CDKN1A compared to empty vector (**h**, **i**), and was induced in cells transfected with CDKN1A-siRNA compared to scramble siRNA (**j**, **k**). *p < 0.05; **p < 0.01; ***p < 0.001

As miR-302d-3p regulates RPE differentiation by targeting p21^Waf1/Cip1^, we next determined whether p21^Waf1/Cip1^ could benefit the regular function of RPE. RPE differentiation relevant markers, including ZO-1, microphthalmia-associated transcription factor (MITF, NP_937802), MERTK, β-Catenin, and Keratin 18, were elevated in hiPSC-RPE cells at 30 dpd overexpressing endogenous p21^Waf1/Cip1^ (Fig. 5a, b). Concomitantly, those markers were reduced in hiPSC-RPE cells with *CDKN1A* knocked down (Fig. 5a, c). Thus, our data suggested a promotive role of *CDKN1A* in RPE differentiation.

We also monitored impacts of p21^Waf1/Cip1^ on RPE proliferation, migration, and cell-cycle progression. As indicated by our findings, rates of both proliferation and migration were downregulated in ARPE-19 cells transfected with AcFlag-CDKN1A (Fig. 5d, f), while were upregulated in cells transfected with CDKN1A-siRNA (Fig. 5e, g), supporting that p21^Waf1/Cip1^ inhibits RPE proliferation and migration. Furthermore, p21^Waf1/Cip1^ overexpression resulted in increased fraction of ARPE-19 cells in S and G2/M phases and decreased fraction in G0/G1 phases (Fig. 5h, i), while knock down of *CDKN1A* showed opposite effects by promoting cell-cycle progression (Fig. 5j, k). Altogether, our findings indicated that p21^Waf1/Cip1^ promotes RPE differentiation, and inhibits its proliferation, migration, and cell-cycle progression.

### MiR-302d-3p induces VEGF-A secretion by RPE cells, and triggers tube formation of HUVECs through regulating of p21^Waf1/Cip1^

**Fig. 6:**
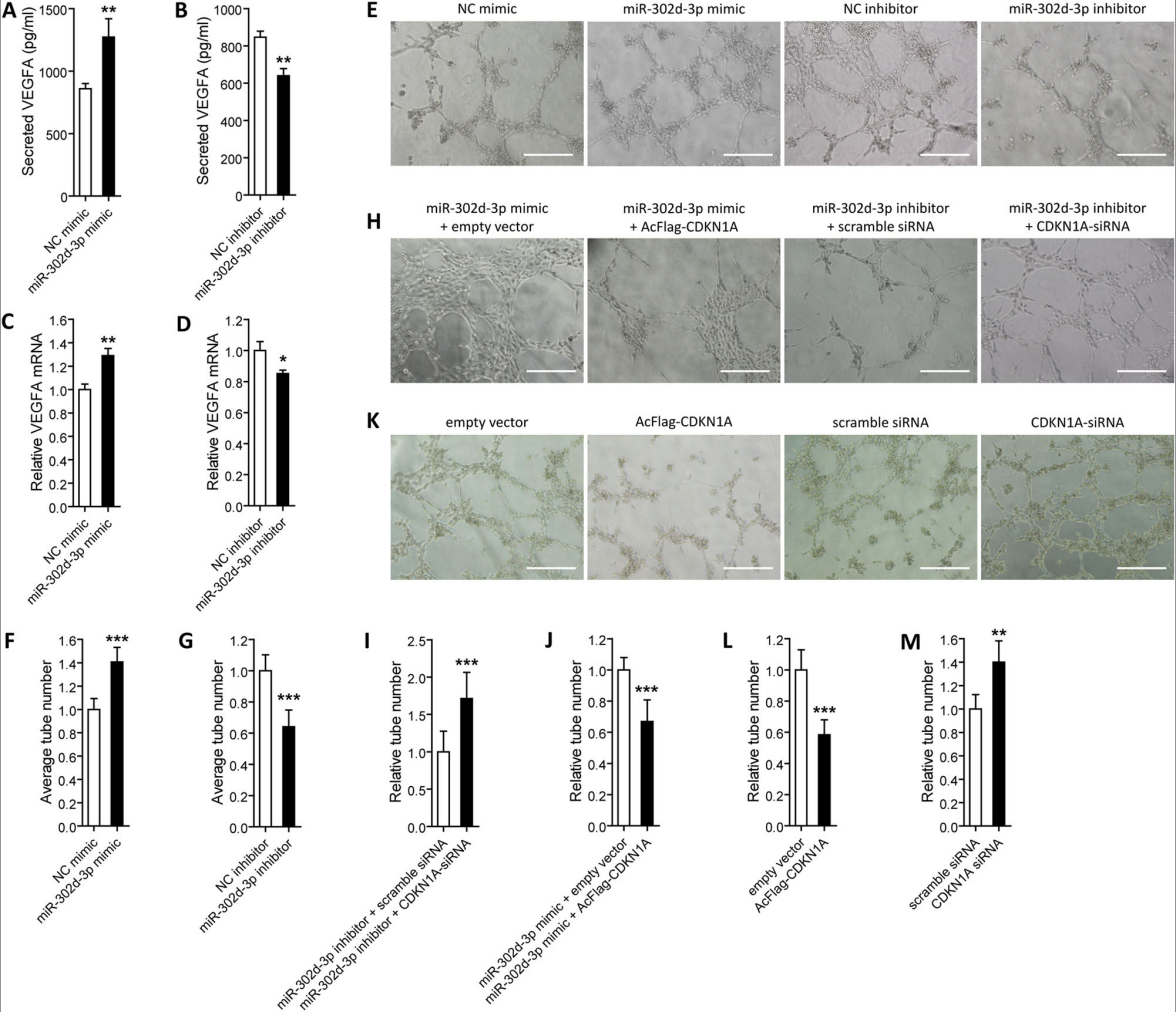
MiR-302d-3p regulates tube formation of HUVECs through p21^Waf1/Cip1^. **a**, **b** Secreted VEGF-A protein were increased in ARPE-19 cells transfected with miR-302d-3p mimic compared to NC mimic (**a**), and were decreased in ARPE-19 cells transfected with miR-302d-3p inhibitor when compared to NC inhibitor (**b**). **c**, **d** *VEGFA* mRNA were increased in ARPE-19 cells transfected with miR-302d-3p mimic when compared to cells transfected with NC mimic (**c**), and were decreased in ARPE-19 cells transfected with miR-302d-3p inhibitor compared to NC inhibitor (**d**). **e**–**g** Tube formation was promoted in HUVECs transfected with miR-302d-3p mimic compared to NC mimic (**e**, **f**), and was inhibited in cells transfected with miR-302d-3p inhibitor compared to NC inhibitor (**e**, **g**). **h**–**j** Tube formation was suppressed in HUVECs transfected with miR-302d-3p mimic plus AcFlag-CDKN1A compared to miR-302d-3p mimic plus empty vector (**h**, **i**), and was induced in cells transfected with miR-302d-3p inhibitor plus CDKN1A-siRNA compared to miR-302d-3p inhibitor plus scramble siRNA (**h**, **j**). **k**–**m** Tube formation was decreased in HUVECs transfected with AcFlag-CDKN1A compared with empty vector (**k**, **l**), and was increased in cells transfected with CDKN1A-siRNA compared to scramble siRNA (**k**, **m**). *p < 0.05; **p < 0.01; ***p < 0.001. Scale bar = 100 μm

As RPE dysfunction or degeneration also contributes to the onset and progression of wet AMD^37,38^, we therefore aimed to tell whether miR-302d-3p participates in CNV generation. Aberrant and redundant VEGF-A secretion in RPE cells triggers CNV formation^39^, we thus first measured expressions of VEGF-A (NP_003367) protein secreted by RPE cells and *VEGFA* (NM_003376) mRNA expressed in RPE cells. Our data suggested that both secreted VEGF-A protein and expressed *VEGFA* mRNA were increased in ARPE-19 cells transfected with miR-302d-3p mimic when compared to cells transfected with NC mimic (Fig. 6a, c), and were decreased in ARPE-19 cells transfected with miR-302d-3p inhibitor when compared to cells transfected with NC inhibitor (Fig. 6b, d). We next performed tube formation assay on HUVECs. We found that miR-302d-3p overexpression induces tube formation of HUVECs (Fig. 6e, f), while miR-302d-3p insufficiency suppresses HUVEC tube formation (Fig. 6e, g). Our data indicated the involvement of miR-302d-3p in triggering abnormal EC behaviors. We next detected whether miR-302d-3p regulates tube formation of HUVECs through p21^Waf1/Cip1^. Our findings suggested that p21^Waf1/Cip1^ overexpression could medicate the promotive effect of miR-302d-3p mimic on tube formation; while silencing of *CDKN1A* suppressed the inhibitory role of miR-302d-3p inhibitor (Fig. 6h–j). Further assessments suggested that tube formation of HUVECs were decreased in cells transfected with AcFlag-CDKN1A, and were increased in cells transfected with CDKN1A-siRNA (Fig. 6k–m). Collectively, these data implied that miR-302d-3p promotes VEGF-A secretion by RPE cells and tube formation of HUVECs by directly targeting p21^Waf1/Cip1^, implying its potential involvement in CNV generation.

### MiR-302d-3p promotes proliferation and migration of HUVECs

**Fig. 7:**
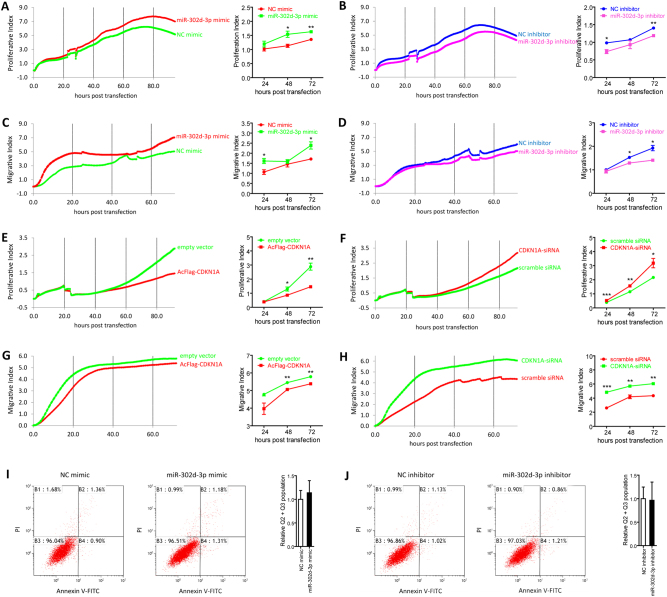
MiR-302d-3p promotes HUVEC proliferation, migration, and cell-cycle progression. **a**, **b** Proliferation was promoted in ARPE-19 cells transfected with miR-302d-3p mimic compared to NC mimic (**a**), and was inhibited in cells transfected with miR-302d-3p inhibitor compared to NC inhibitor (**b**). **c**, **d** Migration was suppressed in ARPE-19 cells transfected with miR-302d-3p mimic compared to NC mimic (**c**), and was promoted in cells transfected with miR-302d-3p inhibitor compared to NC inhibitor (**d**). **e**, **f** Proliferative rates was downregulated in ARPE-19 cells transfected with AcFlag-CDKN1A compared to empty vector (**e**), and was upregulated in cells transfected with CDKN1A-siRNA compared to scramble siRNA (**f**). **g**,** h** Migration was suppressed in ARPE-19 cells transfected with AcFlag-CDKN1A compared to empty vector (**g**), and was promoted in cells transfected with CDKN1A-siRNA compared to scramble siRNA (**h**). **i**, **j** No detectable change in apoptosis was revealed in ARPE-19 cells transfected with miR-302d-3p mimic compared to NC mimic (**i**), nor in cells transfected with miR-302d-3p inhibitor compared to NC inhibitor (**j**). *p < 0.05; **p < 0.01; ***p < 0.001

To better understand the cellular effects of miR-302d-3p/*CDKN1A* axis on HUVEC function, we further assessed whether this axis will affect the proliferation and migration of HUVECs. According to our results, both proliferation and migration were promoted in HUVECs transfected with miR-302d-3p mimic (Fig. 7a, c), while were inhibited in cells transfected with miR-302d-3p inhibitor (Fig. 7b, d). In addition, proliferation and migration rates were reduced in HUVECs overexpressing p21^Waf1/Cip1^ (Fig. 7e, g), while were elevated in cells with p21^Waf1/Cip1^ insufficiency (Fig. 7f, h). No apoptosis was detected in HUVECs transfected with miR-302d-3p mimic or inhibitor when compared with the control group (Fig. 7i, j). Our data suggested that miR-302d-3p shows a promotive role in HUVEC proliferation and migration, further indicating its role in promoting tube formation of HUVEC.

## Discussion

In mature organs and tissues, miRNAs have more essential roles in monitoring cellular stress than primary cellular functions^40^. Essential roles of miRNAs and their processing factors in keeping the survival of RPE cells have been revealed^16,20,41,42^. Thus, investigating the influence of miRNAs on retinal degenerative diseases and seeking for potential treatment are promising and expanding. We have previously identified that, miR-302 members including miR-302d-3p, miR-302a-5p, miR-302a-3p, and miR-302c-5p, were consistently downregulated along with the differentiation of RPE cells^20^. Herein, we select the most declined miR-302d-3p for further analysis, and revealed that miR-302d-3p triggers RPE dedifferentiation, inhibits RPE phagocytosis, and further induces abnormal EC behaviors by targeting p21^Waf1/Cip1^. Dedifferentiation of RPE contributes to the pathogenesis of atrophic AMD, and abnormal choroidal vascular functions associates with exudative AMD^12,20^. Thus, our finding indicates that miR-302d-3p might have a role in the pathogenesis of both atrophic and exudative AMD. Compared to traditional therapeutic strategies, miRNA-based treatments have more advantages in drug efficiency and delivery^16^. Gene therapies targeting miRNAs in treating CNV have also been well developed^43,44^. Therefore, miR-302d-3p inhibitors with high efficiency, including tough decoys (TuDs) and small guide RNA with CRISPR/Cas9 system, are prospective in suspending or even preventing AMD disease course^45,46^.

MiR-302 family includes mature miRNAs encoded by genes *MIR302A*, *MIR302B*, *MIR302C*, and *MIR302D*. These miRNAs are highly conserved, share the same seed region, and only differ from one another by a few nucleotides^47^. Therefore, other miR-302 members might also be involved in this process, and might have very similar functions with miR-302d-3p due to their high sequential similarity and shared seed regions and binding sites with *CDKN1A* 3′-UTR. MiR-302s, crucial for regulating self-renewal and pluripotency of stem cells, are highly expressed in embryonic stem cells (ESCs), iPSC, and during early embryogenesis^48^. MiR-302 is required for embryonic viability, and knocking out of miR-302s would interrupt mammalian embryonic development and cause abnormal eye development^49^. MiR-302 is also important for neural development, including timing of neural differentiation and neural tube closure^49^. However, the precious role of miR-302s in modulating RPE function and CNV formation has never been elucidated. We here find that miR-302d-3p contributes to the pathogenesis of both atrophic and exudative AMD by promoting RPE dedifferentiation, triggering abnormal EC behavior, inducing RPE apoptosis, and inhibiting RPE phagocytosis. Our data also reveal that miR-302d-3p triggers RPE proliferation, migration, and cell-cycle progression. The effects of miR-302s on cell proliferation are variable depending on different cell types. MiR-302s are reported to induce proliferation in hASDCs, and suppress proliferation in various cancer cells^35,50–52^.

The JNK signaling pathway regulates various biological processes like cell proliferation, migration, survival, and cytokine production^5^. JNK has crucial roles in ocular and other degenerative diseases^53^. Reactive oxygen species (ROS), which is involved in AMD disease cause, could activate JNK and cause cell death^54^. Reportedly, activation of the JNK pathway is involved in inflammation and VEGF production, and JNK inhibition leads to a decrease in apoptosis and reduction of CNV generation^5^. In this study, we first reveal c-Jun as a potential upstream transcript factor for *MIR302D*, offering a unique and alternative explanation for the involvement of JNK in AMD pathogenesis.

MiR-302s have many cell-cycle targets, such as *OCT4*/*SOX2*, *NR2F2*, *CCND1*, *CDK2*, and *CDKN1A*^23,35,52,55^. Previous studies have confirmed that miR-302s, including miR-302d-3p, could directly bind to the 3′-UTR of *CDKN1A* mRNA in various cell types^35,56,57^. P21^Waf1/Cip1^, protein encoded by *CDKN1A*, is a cyclin-dependent kinase inhibitor that prevents cells from going through the G1/S phase checkpoint^36^. Increased p21^Waf1/Cip1^ expression in RPE cells inhibits its proliferation and migration^36^. In this study, our data support that miR-302d-3p modulates RPE function and EC behavior by targeting p21^Waf1/Cip1^. We also reveal that p21^Waf1/Cip1^ promotes RPE differentiation, inhibits RPE proliferation, migration, cell-cycle progression, and facilitates EC function.

In conclusion, our study implies that miR-302d-3p promotes RPE dedifferentiation and induces abnormal EC function by targeting p21^Waf1/Cip1^, which might contribute to the pathogenesis of both atrophic and exudative forms of AMD. MiR-302d-3p inhibitors are prospective therapeutic options for AMD treatment. However, in vivo animal studies are still warranted to better illustrate whether miR-302d-3p over-dosage could directly cause AMD and the underlying precise mechanism.

## Materials and methods

### Mimics, inhibitors, siRNA, and plasmids

Human miR-302d-3p mimic and inhibitor, together with negative control (NC) mimic and inhibitor, were purchased from GenePharma Co., Ltd (Shanghai, China). Scramble siRNA (NControl_0518) and CDKN1A-siRNA were purchased from Ribobio (Guangzhou, China). Sequences of mimics, inhibitors, and siRNAs were detailed in Supplementary Table S1. Open reading frame sequence of the *CDKN1A* gene was synthesized, amplified, and inserted into the expression vector pCMV-C-Flag (Beyotime). *Bam*HI and *Xba*I restriction sites were used to generate the AcFlag-CDKN1A plasmid with primers listed in Supplementary Table S2. A 63 bp fragment from the 3′-UTR of the *CDKN1A* gene containing its binding region with miR-302d-3p was synthesized and cloned into the pGL3-Promoter Vector (Promega, Madison, WI, USA) using *Nhe*I and *Xho*I restriction sites to construct the recombinant plasmids *CDKN1A*^WT^ and *CDKN1A*^MU^. The *CDKN1A*^MU^ plasmid covered 11 mutated nucleotides located in the core binding region of *CDKN1A* as shown in Fig. 3j. Constructed plasmids were sequenced and confirmed using Sanger sequencing.

### **C**ell culture and transfection

HiPSC were cultured and differentiated into RPE cells as detailed previously^20^. Both ARPE-19 cells and HUVECs were purchased from American Type Culture Collection (ATCC). ARPE-19 cells were maintained in Dulbecco’s Modified Eagle (DME)/F12 medium supplemented with 10% fetal bovine serum (FBS; Invitrogen, Carlsbad, CA, USA), penicillin (100 U/ml) and streptomycin (100 g/ml) at 37 °C, 5% CO_2_. HUVECs were cultured in F12 medium supplemented with 10% FBS, 0.05 mg/ml endothelial cell growth supplement (BD Biosciences, Palo Alto, CA, USA), penicillin (100 U/ml), and streptomycin (100 g/ml) at 37 °C, 5% CO_2_. Complete medium was short for supplemented culture medium in the following text. For transfection assay, hiPSC-RPE at 30 dpd, ARPE-19 cells and HUVECs were initially seeded into 6-well templates. Cells were transfected with 100 pmol mimic/inhibitor/siRNA, and/or 4 μg expression vector at 50–60% confluence using Lipofectamine^TM^ 2000 transfection reagent (Invitrogen) per the manufacturers’ protocol.

### RNA extraction, RT-PCR, and real-time PCR

Both hiPSC-RPE at 30 dpd and ARPE-19 cells were harvested at 48 h post transfection for RNA isolation. Total RNA was extracted with TRIzol reagent (Invitrogen) as indicated previously^58,59^. RNA concentration and quality were measured using Nano-Drop ND-1000 spectrophotometer (Nano-Drop Technologies, Wilmington, DE, USA). cDNA was then synthesized for mRNA with a PrimeScript RT Kit (Takara, Otsu, Shiga, Japan), and was generated for miRNA by stem-loop reverse transcription with oligo-dT primers (RiboBio, Guangzhou, China). Real-time PCR for both mRNA and miRNA was performed using FastStart Universal SYBR Green Master (ROX; Roche, Basel, Switzerland) with the StepOne Plus Real-time PCR System (Applied Biosystems, Darmstadt, Germany). Primers were provided in Supplementary Table S2. Human Glyceraldehyde-3-phosphate dehydrogenase (*GAPDH*; NM_002046) and *U6* gene expressions were analyzed in parallel for normalization of mRNA and miRNA expressions, respectively.

### Immunoblotting and immunofluorescence

ARPE-19 cells were planted into 6-well plates for immunoblotting, and were grown on 8-well chamber slides (Millipore, Billerica, MA, USA) for immunofluorescence. Cells were maintained in complete medium, and were harvested at 72 h post transfection. Immunoblotting and immunofluorescence were conducted using a previously described protocol^60,61^. Antibodies were detailed in Supplementary Table S3. ImageJ software (https://imagej.nih.gov/ij/index.html) was applied to determine and quantify protein expressions.

### Analysis of phagocytosis

Carboxylate-modified polystyrene latex beads (1 μm in diameter; Sigma) with yellow-green fluorescence (emission maximum: 515 nm) were used for phagocytosis analysis. ARPE-19 cells were grown and transfected on 8-well chamber slides (Millipore). At 48 h post transfection, cells were then incubated with phosphate buffered saline (PBS) diluted fluorescent beads (70 beads per cell) at 37 °C for 12 h. After the incubation, cells were washed with PBS for three times to stop the phagocytosis, treated with 0.2% trypan blue for 10 min (min) to quench extracellular fluorescence, and fixed in 4% paraformaldehyde (PFA) for 15 min. Cell nuclei were then counterstained by 4′, 6-diamidino-2-phenylindole (DAPI; Sigma) for 5 min. Images were collected with an Olympus IX70 confocal laser-scanning microscope (Olympus, Tokyo, Japan). ImageJ software was used to quantify fluorescence.

### **A**poptosis assay

ARPE-19 cells and HUVECs at 48 h post transfection were collected, washed with PBS, suspended in staining buffer, and incubated with Annexin-V-FITC (R&D, New Jersey, USA) and PI (R&D) per the manufacturers’ protocol. Flow-cytometric analysis was subsequently performed to identify apoptotic cells with a gallios flow cytometry (Beckman Coulter, Brea, USA). A total of 10,000 living cells were collected for each sample. Three groups of untreated ARPE-19 cells and HUVECs were included for scatter gating: (1) Unstained cells for cell selection and adjustment of photomultiplier voltage; (2) Annexin-V-FITC stained only cells for adjustment of the FITC channel; (3) PI stained only cells for adjustment of the phycoerythrin channel. Data were then displayed as two-color dot plot with Annexin-V-FITC (X axis) vs. PI (Y axis). Annexin-V positive cells were recognized as apoptotic cells.

### Monitoring cell proliferation and migration

We used xCELLigence system E-Plate (Roche) to monitor the proliferation and migration rates of ARPE-19 cells and HUVECs according to the manufacturer’s protocol. To measure the proliferation rates, 5000 cells were planted into each well of the E-Plate, and were transfected at 24 h post plantation. For migration analyses, 40,000 cells were seeded into each well right after transfection. All cells were maintained in complete medium. Impedance values were automatically monitored by the xCELLigence system and expressed as a cell index value.

### Cell-cycle analysis

ARPE-19 cells from different transfected groups were harvested and fixed with 70% ethanol overnight. After extensive washing with PBS, cells were then suspended in PBS containing propidium iodide (Sigma), and incubated for 30 min before flow-cytometric analysis. Acquired data were then analyzed using the kaluza for gallios software (Beckman Coulter). A total of 10,000 cells from each sample were measured to modulate the cell-cycle.

### Luciferase reporter assay

Luciferase reporter assay was performed according to a previously defined protocol^20,61^. Briefly, cells were seeded into 24-well plates and transfected with 16 ng cytomegalovirus-Renilla (Promega), 20 pmol miR-302d mimic or NC mimic, and 800 ng *CDKN1A*^WT^ or *CDKN1A*^MU^. Cells were collected 72 h post transfection for luciferase activities measurement using the dual luciferase system (Promega) with a GloMax-96 luminometer. Renilla luciferase activities were taken as internal standard indicators for transfection efficiency. Firefly luciferase activities were further normalized to Renilla luciferase activities.

### Enzyme-linked immunosorbent assay (ELISA)

To determine the secretion of VEGF-A, ARPE-19 were planted onto 6-well transwell plates (Corning) precoated with matrigel (1:30 diluted in DME/F12 medium; BD Biosciences, San Jose, CA, USA). The culture medium was collected and changed every day post transfection. Collected medium was used to determine the expression level of VEGF-A using a commercial human VEGF-A ELISA kit (Beijing 4A Biotech Co., Ltd, Beijing, China) per the manufacturer’s protocol.

### Tube formation assay

For detection of capillary-like structure formation, transfected HUVECs were planted onto growth factor-reduced matrigel (BD Biosciences) in a 24-well plate. Cells were observed at 24 h post plantation with a bright-filed microscope. ImageJ software was used to analyze and quantify tube number.

### Statistics

We used GraphPad Prism (version 4.0; GraphPad Software, San Diego, CA, USA) for statistical analysis. Student’s *t*-test was applied for comparison between different groups. All experiments were performed in triplicates with data averaged. Data were presented as mean ± standard deviation (SD). *p* value < 0.05 was taken as statistically significant.

## Electronic supplementary material

Supplementary Table S1

Supplementary Table S2

Supplementary Table S3
