# Supplementary material for: c-Jun-mediated microRNA-302d-3p induces RPE dedifferentiation by targeting p21Waf1/Cip1
Source: Cell Death Dis. 2018 Apr 18;9(5):451. doi: 10.1038/s41419-018-0481-5 (PMC5906557; doi:10.1038/s41419-018-0481-5)
Supplement: Supplementary file 1 — Supplementary Table S1 [file 41419_2018_481_MOESM1_ESM.docx]

| **Table S1** Sequences of mimics/inhibitors/siRNAs | |
| --- | --- |
| Mimic/Inhibitor/siRNA | Sequence (5'→3') |
| NC-mimic | UUCUCCGAACGUGUCACGUTT |
| NC-inhibitor | CAGUACUUUUGUGUAGUACAA |
| miR-302d-3p mimic | UAAGUGCUUCCAUGUUUGAGUGU |
| miR-302d-3p inhibitor | ACACUCAAACAUGGAAGCACUUA |
| CDKN1A-siRNA | GGACCTGTCACTGTCTTGT |
| *CDKN1A*^WT^ | GCTAGCTTCTTTTTCATTTGAGAAGTAAACAGATGGCACTTTGAAGGGGCCTCACCGCTCGAG |
| *CDKN1A*^MU^ | GCTAGCTTCTTTTTCATTTGAGAAGTCCCACGATGTACAGGTGAAGGGGCCTCACCGCTCGAG |
|  | *mutated spot* *mutated spot* |
