# Supplementary material for: c-Jun-mediated microRNA-302d-3p induces RPE dedifferentiation by targeting p21Waf1/Cip1
Source: Cell Death Dis. 2018 Apr 18;9(5):451. doi: 10.1038/s41419-018-0481-5 (PMC5906557; doi:10.1038/s41419-018-0481-5)
Supplement: Supplementary file 2 — Supplementary Table S2 [file 41419_2018_481_MOESM2_ESM.docx]

| **Table S2** Primers used in this study | | |
| --- | --- | --- |
| Gene/Plasmid | Forward primer (5'→3') | Reverse primer (5'→3') |
| For expression study in human cell line | | |
| *GAPDH* | CAGCCTCAAGATCATCAGCA | TGTGGTCATGAGTCCTTCCA |
| *RPE65* | TACAGAAAGCACTGAGTTGAGC | CCATTTAGTAAGTCCACATTCATTTCC |
| *RLBP1* | GCTGCTCAGAGGCTATGTGA | TGCCTGCAAGATCTCATCAA |
| *MERTK* | AGTGCAGGGATTTCCAAAGA | GGGGCATAATCTACCCAACC |
| *BEST1* | CCTGCTGAACGAGATGAACA | CCACAGTCACCACCTGTGTA |
| *CTNNB1* | TGGACCCCAAGCTTTAGTAAAT | TGGATCTGTCAGGTGAAGTCC |
| *TJP1* | CTTCCAGAACCAAAGCCTGT | ATGCTGGGCCGAAGAATC |
| *MITF* | AGCGTCCTGTATGCAGATGG | CCGAGACAGGCAACGTATTT |
| *PAX6* | ACCGGTTTCCTCCTTCACAT | GGGTTGCATAGGCAGGTTAT |
| *LRAT* | TCAGACCTACCAGTTCTGCAA | CAAACAGGGTCACCGACTG |
| *KRT18* | GGAGACTTCAAACTCCAGGATG | TCTGTGCTTGAGGACACAAC |
| *CDKN1A* | TTTGTCACCGAGACACCACT | CAGCAGAGCAGGTGAGGTG |
| For plasmid construction | | |
| Ac-Flag-CDKN1A | GGATCCATGTCAGAACCGGCTGGGGATGTC | TCTAGAGGGCTTCCTCTTGGAGAAGATCAGC |
|  | *BamHI* | *XbaI* |
| Italic font indicates the genes. | | |
