# Supplementary material for: c-Jun-mediated microRNA-302d-3p induces RPE dedifferentiation by targeting p21Waf1/Cip1
Source: Cell Death Dis. 2018 Apr 18;9(5):451. doi: 10.1038/s41419-018-0481-5 (PMC5906557; doi:10.1038/s41419-018-0481-5)
Supplement: Supplementary file 3 — Supplementary Table S3 [file 41419_2018_481_MOESM3_ESM.docx]

| **Table S3** Antibodies used in this study | | |  |
| --- | --- | --- | --- |
| Anti-protein | Host | Dilution and Application | Supplier |
| ZO-1 | Rabbit | 1:200, Immunoblotting; 1:100, Immunostaining | Invitrogen |
| β-Catenin | Rabbit | 1:1000, Immunoblotting | Cell Signaling Technology |
| GAPDH | Rabbit | 1:5000, Immunoblotting | Bioworld |
| Keratin 18 | Rabbit | 1:10000, Immunoblotting | Abcam |
| p21^Waf1/Cip1^ | Rabbit | 1:1000, Immunoblotting | Cell Signaling Technology |
| MITF | Rabbit | 1:1000, Immunoblotting | Cell Signaling Technology |
| MERTK | Rabbit | 1:1000, Immunoblotting | Cell Signaling Technology |
